# Equivalence between FTP and RCP: Assessment of association, agreement and sex effect

**DOI:** 10.1016/j.clinsp.2026.100977

**Published:** 2026-04-29

**Authors:** Paulo Engelke, Lavínia Vivan, Vinícius Ribeiro dos Anjos Souza, Claudio Andre Barbosa de Lira, Katja Weiss, Thomas Rosemann, Beat Knechtle, Marilia Santos Andrade

**Affiliations:** a Post-Graduate Program in Translation Medicine, Universidade Federal de São Paulo, São Paulo, SP, Brazil; b Human and Exercise Physiology Division, Faculty of Physical Education, Universidade Federal de Goiás, Goiânia, GO, Brazil; c Institute of Primary Care, University of Zurich, Zurich, Switzerland; d Medbase St. Gallen Am Vadianplatz, St. Gallen, Switzerland; e Department of Physiology, Universidade Federal de São Paulo, São Paulo, SP, Brazil

**Keywords:** Athletes, Maximal metabolic steady state, VO_2_max, Cycling, Triathlon

## Abstract

**Objectives:**

Functional Threshold Power (FTP) is widely used for performance evaluation and training prescription in endurance sports. Although FTP is strongly associated with power at the Respiratory Compensation Point (RCP), its interchangeability remains debated. This study examined the relationship and agreement between FTP and RCP in amateur triathletes and tested whether sex influences this relationship.

**Methods:**

Forty-nine amateur triathletes (male and female) participated. Each athlete completed a maximal cardiorespiratory cycling test and an FTP test, separated by at least 72 h. Linear regression models were used to test the association between FTP and RCP. A regression model including the interaction term (FTP × sex), equivalent to an ANCOVA framework, was applied to assess sex effects on the FTP-RCP relationship. Agreement between methods was assessed using Bland-Altman analysis. Two-way analysis of variance (ANOVA) was performed to examine the effects of sex, test type, and their interaction on absolute power (W), power relative to total body mass, and power relative to lean mass.

**Results:**

FTP and RCP were strongly correlated (*r* = 0.916; p < 0.001). The interaction term FTP × sex was not significant (β = -0.135, p = 0.524), indicating that the regression slopes do not differ between males and females. Thus, the relationship between FTP and RCP was statistically similar across sexes. However, Bland-Altman analysis revealed wide limits of agreement, indicating poor interchangeability between FTP and RCP at the individual level.

**Conclusions:**

FTP is strongly associated with RCP, and the relationship is independent of sex. Despite this association, FTP and RCP are not interchangeable measures. Caution is warranted when using FTP as a direct surrogate for RCP in training prescription and performance assessment.

## Highlights

• FTP strongly correlates with power at the respiratory compensation point.
• The FTP-RCP relationship is consistent in male and female triathletes.
• FTP and RCP show poor agreement and should not be used interchangeably.
• FTP consistently underestimates the power associated with RCP.
• Sex differences disappear when power is normalized to lean mass.

## Introduction

Endurance performance in activities such as running, cycling, and triathlon is determined by three key physiological variables: Maximum Oxygen uptake (V̇O_2_max), the percentage of V̇O_2_max that can be sustained, and movement economy.^1^ These parameters can be assessed through respiratory gas analysis using a maximal cardiorespiratory ergometer test.^2^

To evaluate the percentage of V̇O_2_max that an athlete can sustain, the Maximal Metabolic Steady State (MMSS) has been suggested, which is a physiological concept that represents the highest exercise intensity at which all physiological variables (including VO_2_, lactate, and ventilation) remain in a steady state. Regarding the blood lactate concentration, the Maximal Lactate Steady State (MLSS) has been proposed as a substitute measure for the MMSS. It is defined as the highest exercise intensity at which blood lactate concentration remains stable (variation ≤1 mmol/L) during prolonged exercise (typically 30-minutes), and it represents the threshold between the heavy and severe intensity domains, and is classically considered the best marker of the upper limit of sustainable exercise without progressive accumulation of metabolic fatigue.^3^^,^^4^ In the same direction, the Respiratory Compensation point, which is identified during incremental tests as the point where hyperventilation occurs disproportionately to the increase in CO_2_ production, reflecting the onset of metabolic acidosis not compensated by buffer systems, is also often used as an indirect marker of MLSS/MMSS as it occurs at intensities close to those of the MLSS.^3^^,^^5^ RCP also serves as a physiological boundary between heavy and severe exercise intensity domains.^6^ However, conducting these tests to identify MLSS or RCP requires specialized equipment, trained personnel, and significant time, which limits its accessibility.^7^

As an alternative method for estimating the highest exercise intensity at which physiological variables remain in a steady state, the Functional Threshold Power (FTP) test has been proposed. Allen and Coggan defined FTP as the mean power output a cyclist can sustain in a quasi-steady state for 60 min.^8^ Since maintaining an all-out effort for this duration is challenging for amateur athletes, a modified protocol has been suggested: a 20 min maximal effort, with FTP estimated as 95% of the mean power output during this period. FTP is widely used in cycling and triathlon training for assessing exercise capacity, determining training zones, and prescribing workouts.^7^

Although FTP and RCP are positively correlated, the extent to which these measures are interchangeable remains a subject of debate.^9^^,^^10^ However, most of these studies were conducted with male athletes, and even when female athletes were included, sex was not considered as a variable to be analyzed. Furthermore, sex-based differences in endurance performance and RCP suggest that the strength of the relationship between FTP and RCP may vary between men and women.^11^ Studies on this topic have focused primarily on male subjects, leaving the potential influence of sex largely unexplored.

Given the inconsistent evidence regarding the equivalence of FTP and RCP and the lack of research examining sex-based differences, this study aimed to test whether sex moderates the FTP-RCP relationship. Moreover, the study also aimed to investigate whether the FTP test could replace the incremental test for determining power at RCP. The study hypothesized that sex would moderate the relationship between FTP and RCP, and FTP and RCP would not yield equivalent values.

## Materials and methods

### Ethical approval

This was a cross-sectional study that followed the STROBE Statement. All experimental procedures were approved by the Human Research Ethics Committee of an institution affiliated with one of the authors and were conducted in accordance with the principles outlined in the Declaration of Helsinki. All participants provided written informed consent before enrollment in the study. CAAE: 66,648,122.3.0000.5505.

### Participants

This study was conducted at the Exercise Physiology Department of the Federal University of São Paulo, located at the Training and Research Olympic Center in São Paulo, Brazil.

A total of 49 amateur triathletes (25-males and 24-females) participated in the study. The athletes were recruited from triathlon training clubs that use the Olympic Training and Research Center area to conduct their activities. Their anthropometric and V̇O_2_max data are presented in Table 1. The inclusion criteria were as follows: at least 1 year of triathlon experience and engagement in a minimum of five training sessions per week. The exclusion criteria included inability to complete any of the tests, pain experienced during the tests, and the presence of any chronic disease.

**Table 1:** General characteristics of participants.

Variable	Male (n = 25) Mean ± SD	Female (n = 24) Mean ± SD	p-value	Effect size	95% CI
Age (yr)	33.4 ± 8.9	39.2 ± 7.4	0.018	0.70	0.52 to 0.94
Body mass (kg)	76.3 ± 9.3	63.2 ± 6.9	<0.001	1.60	1.20 to 2.10
Height (cm)	1.77 ± 0.06	1.66 ± 0.07	<0.001	1.70	1.30 to 2.30
V̇O_2_max (mL/kg/min)	51.8 ± 8.0	44.6 ± 4.4	<0.001	1.11	1.00 to 1.20

95% CI, 95% Confidence Interval; V̇O_2_max, Maximal Oxygen uptake.

Participants completed two different tests under similar environmental conditions and at the same time of day, with a minimum interval of 72 h between sessions. On the first day, body composition, body mass, and height were assessed, followed by a maximal cardiorespiratory cycle ergometer test. On the second day, the participants performed an FTP test.

### Anthropometric and body composition assessments

Height was measured using a stadiometer, and body mass was recorded using a scale (Filizola, Brazil). Body composition was assessed using dual-energy X-Ray absorptiometry (DXA; GE Healthcare, USA), a validated method with a typical measurement error of 1.3% for lower limb lean mass.^12^ Participants were instructed to wear comfortable, metal-free clothing. During the scan, all participants lay supine and centrally aligned on the DXA bed, maintaining a 10 cm distance between the feet and a 5 cm distance between the hands and torso. All tests were performed by the same experienced examiner.

### Maximal cardiorespiratory cycle ergometer test

The maximal cycle ergometer test was performed using a cycle ergometer (Excalibur Sport 2019, Lode, Netherlands) and a respiratory gas exchange analyzer (Quark, COSMED, Italy). The respiratory analyzer has demonstrated high accuracy, with an acceptable margin of error.^13^ Respiratory data were collected breath-by-breath and averaged every 20 s for analysis. Calibration procedures were conducted before each test according to the manufacturer’s instructions.

The test protocol consisted of a 3 min warm-up, followed by a continuous incremental load increase of 25 W per minute until volitional exhaustion. V̇O_2_max was determined based on a plateau in oxygen uptake, defined as an increase of <1.5 mL/kg/min despite an increase in workload.^14^ If no plateau was observed, V̇O_2_max was determined using the following criteria: 1) Respiratory Exchange Ratio (RER) >1.15; 2) Achievement of ≥95% of the age-predicted maximal Heart Rate (HRmax) calculated as 207 − (0.7 × age), and 3) A maximal rating of perceived exertion on the Borg scale.^15^

The RCP was identified based on the inflection point of the Ventilatory Equivalent for Carbon Dioxide (V̇E/V̇CO_2_) curve and the decrease in end-tidal carbon dioxide pressure.^2^ Data were analyzed based on absolute power, power relative to body mass, and power relative to lean body mass.

### Functional threshold power test

On the second day, participants completed the FTP test. The ergometer was configured in its linear mode, which permitted participants to self-regulate cadence and resistance to simulate the gearing system and real road conditions. The test protocol began with a 10-min self-paced warm-up, followed by four 20-second maximal sprints, each separated by a 1 min rest period. After the warm-up, participants rested for 5 min before beginning the 20 min maximal cycling test. The warm-up protocol, originally proposed by Allen and Cogan, stipulated a 46-minute warm-up including a 5-minute Time Trial (5TT).^8^ However, it has been previously demonstrated that this 5TT caused a significant increase in blood lactate concentration,^16^ which compromised performance in the subsequent Functional Threshold Power test.^17^ For this reason, a warm-up excluding the 5-minute time trial was selected. The selected warm-up protocol has been chosen because an active warm-up, consisting of a brief aerobic portion (5‒15 min) and completion of 4‒5 activation sprints has been suggested to improve performance.^18^

Participants were instructed to maintain the highest possible average power output throughout the test. FTP was determined as 95% of the mean power output recorded during the 20 min effort.^8^ Data analysis considered absolute power, power relative to body mass, and power relative to lean body mass.

### Data analysis

Based on a priori sample size calculation, the authors determined the minimum number of participants needed to detect a moderate correlation. The calculation was performed using a moderate effect size (0.5), an alpha error of 0.05, and a power of 80%, resulting in a minimum requirement of 21 volunteers for each sex. As the sample size estimation was specifically designed for correlation analyses, the ANOVA-equivalent and regression analyses are presented as post hoc and should be interpreted accordingly.

All values are presented as means, standard deviations, and effect sizes. Normality and homogeneity of variance were confirmed using the Shapiro-Wilk test and Levene’s test, respectively.

Anthropometric characteristics were compared between male and female participants using a Student’s *t*-test. Effect size magnitudes were classified using Cohen’s *d* as follows: < 0.2 (no effect), 0.2–0.49 (small effect), 0.5–0.79 (medium effect), 0.8–1.29 (large effect), and ≥ 1.3 (very large effect).^19^

The Pearson correlation coefficient was used to assess the association between FTP and RCP. Correlation strength was classified as weak (0.10–0.30), moderate (0.31–0.70), and strong (> 0.71).

Although sex-specific correlations were initially explored for descriptive purposes, the primary analytical approach was based on a regression model equivalent to ANCOVA, which allows the evaluation of the association between FTP and power at RCP while formally testing the effect of sex as a covariate and the presence of an interaction. This approach provides a more appropriate framework for assessing sex invariance of the FTP-RCP relationship. Therefore, to evaluate whether sex influenced the relationship between FTP and RCP, an additional linear regression model was performed, including an interaction term (FTP × sex). In this model, RCP (W) was entered as the dependent variable, FTP (W) as the continuous predictor, and sex (male = 0; female = 1) as a categorical moderator. The interaction term allowed testing whether the regression slopes differed between males and females, providing a formal assessment of slope and intercept heterogeneity between groups. This analytical approach is statistically equivalent to an analysis of covariance (ANCOVA) for comparing regression lines between groups.

To examine the effects of sex (male vs. female), test type (power at FTP vs. power at RCP), and their interaction on absolute power, power relative to total body mass, and power relative to lean mass power, a two-way analysis of variance (ANOVA) was conducted. When a significant main or interaction effect was detected, *post hoc* comparisons were performed using Sidak’s test. Agreement between FTP and RCP was assessed using Bland-Altman plots.

The significance level was set at p < 0.05. All statistical analyses were performed using SPSS version 26.0 (SPSS, Inc., Chicago, Illinois).

## Results

**Fig. 1:**
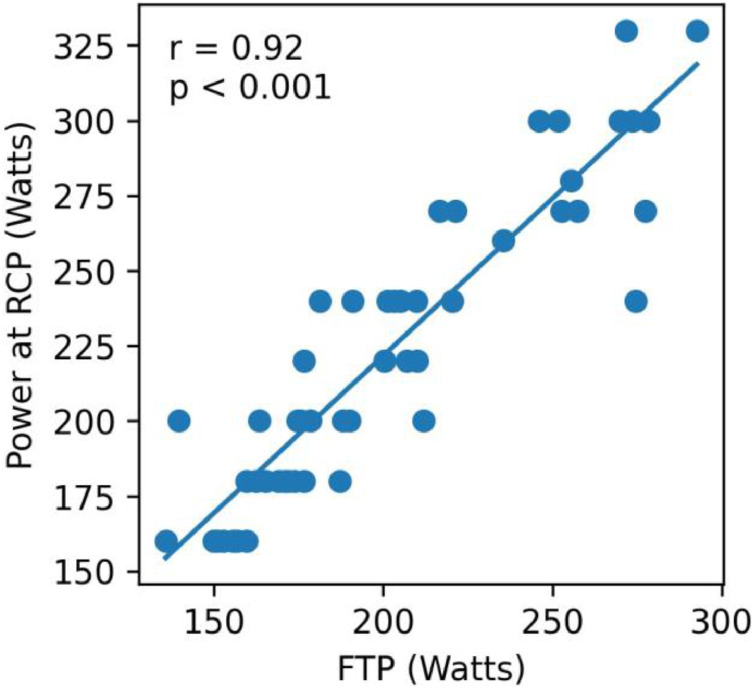
Association between power values at the respiratory compensation point and functional threshold power test.

**Fig. 2:**
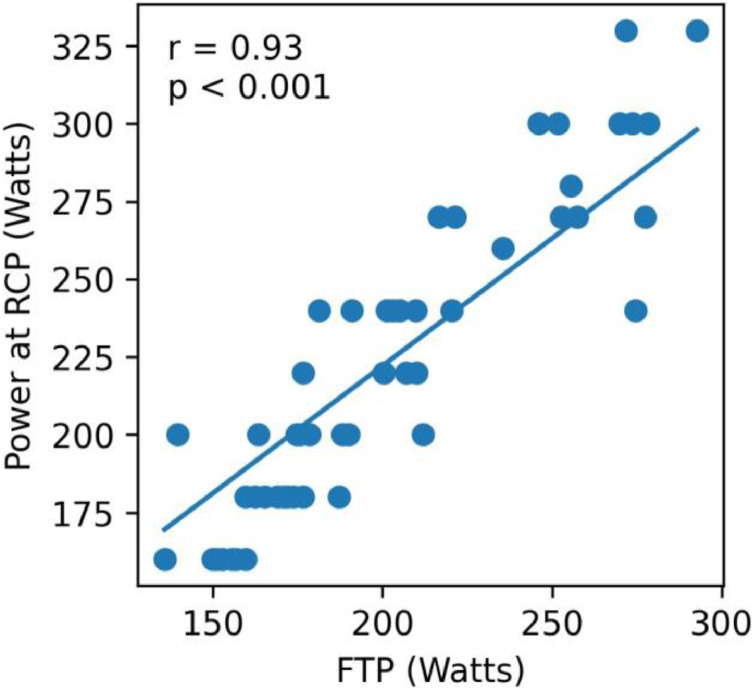
Association between Functional Threshold Power (FTP) and power at Respiratory Compensation Point (RCP). The solid line represents the linear regression adjusted for sex (included as a covariate). The FTP × sex interaction was not significant, indicating that the association between FTP and power at RCP does not differ between males and females.

An initial analysis was performed to evaluate the association between power output determined by the FTP test and power output at the RCP across the entire sample. The results indicated an overall strong correlation when considering men and women together (*r* = 0.916, p < 0.001; Fig. 1). Moreover, the influence of sex on the FTP-RCP relationship was also tested. The linear regression model, including the interaction term (FTP × sex), showed that sex did not significantly modify the relationship between FTP and RCP. The interaction term was not significant (β = −0.135, p = 0.524), indicating that the regression slopes were statistically similar between males and females. Thus, the association between FTP and RCP was independent of sex (Fig. 2). Linear regression model results predicting RCP with FTP, sex, and FTP × sex interaction were presented in Table 2.

**Table 2:** Linear regression model predicting RCP with FTP, sex, and FTP × sex interaction.

Predictor	β coefficient	Standard Error	95% Confidence Interval	p-value
Intercept	63.94	24.17	15.30 to 112.58	0.011
FTP	0.85	0.10	0.65 to 1.06	<0.001
Sex	−1.26	39.78	−81.33 to 78.81	0.975
FTP × Sex	−0.14	0.21	−0.56 to 0.29	0.524

Values are presented as unstandardized regression coefficients (β). Sex was coded as 0 = Male and 1 = Female. FTP, Functional Threshold Power; RCP, Respiratory Compensation Point. Confidence Intervals correspond to 95%.

**Fig. 3:**
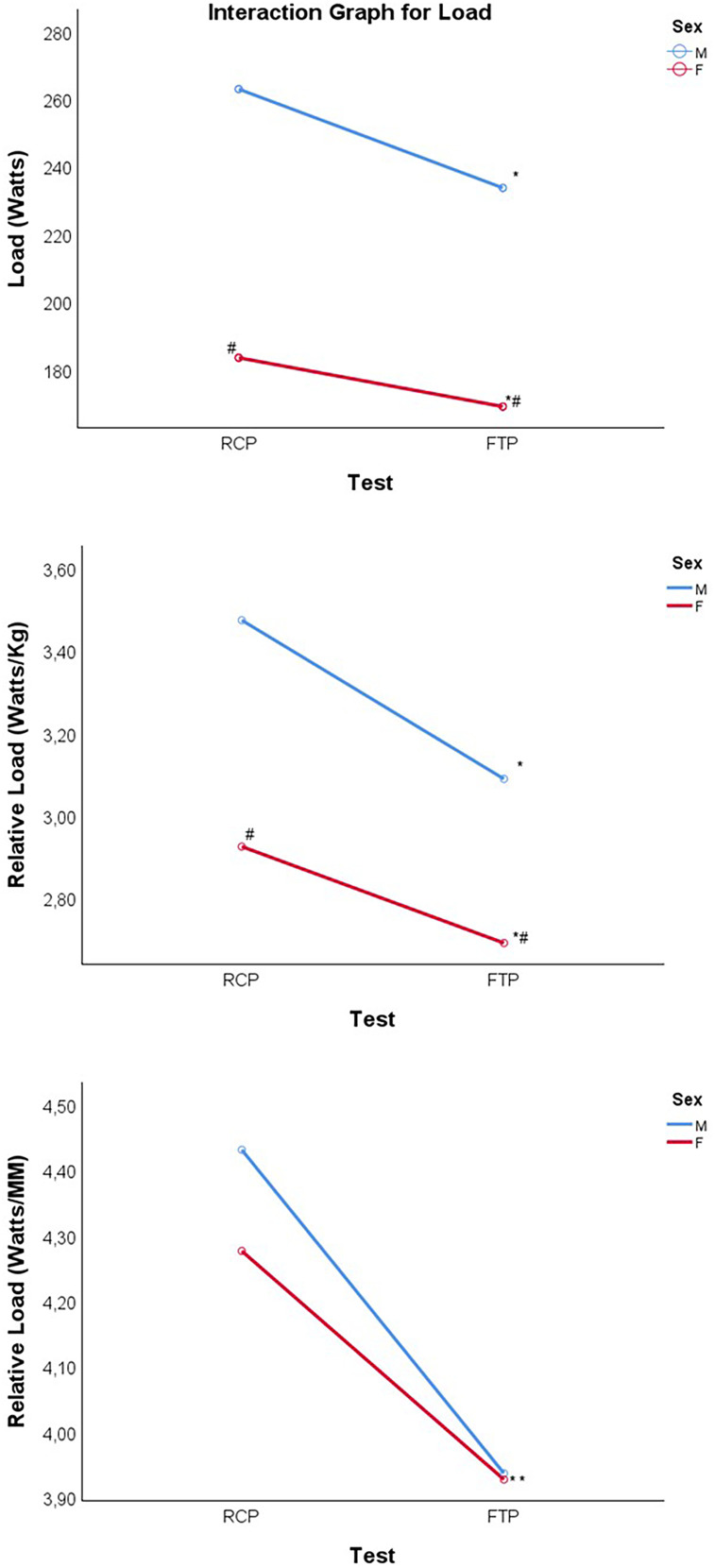
Power values for RCP and FTP in male and female athletes.

The two-way ANOVA revealed a significant effect of test type on absolute power output (W) (*F*(1,47) = 65.735; p < 0.001), a significant effect of sex (*F*(1,47) = 81.627; p < 0.001), and a significant interaction between test type and sex (F(1,47) = 7.541; p = 0.009; Table 3 and Fig. 3).

**Table 3:** Two-way ANOVA for power, relative power (body mass and lean mass), and their interaction with FTP, RCP, and sex.

	Test	Men (n = 25) Mean ± *SD*	Women (n = 24) Mean ± *SD*	ANOVA	*F*-value	p-value	Power
**Power (W)**	RCP	262.80 (±37.25)	183.33 (±19.26)^b^	Test effect	65.735	<0.001	1.000
	FTP	233.59 (±36.29)^a^	168.90 (±36.29)^a^^,^^b^	Sex effect	81.627	<0.001	1.000
	Difference (RCP−FTP)	29.21 (±21.50)	14.43 (±15.58)	Interaction effect	7.541	0.009	0.767
**Power relative to body mass (W/kg)**	RCP	3.472 (±0.51)	2.924 (±0.38)^b^	Test effect	59.804	<0.001	1.000
	FTP	3.088 (±0.53)^a^	2.691 (±0.35)^a^^,^^b^	Sex effect	14.954	<0.001	0.966
	Difference (RCP−FTP)	0.384 (±0.29)	0.234 (±0.26)	Interaction effect	3.554	0.066	0.455
**Power relative to lean mass (W/LMkg)**	RCP	4.430 (±0.51)	4.276 (±0.51)	Test effect	58.516	<0.001	1.000
	FTP	3.937 (±0.53)^a^	3.927 (±0.36)^a^	Sex effect	0.417	0.521	0.097
	Difference (RCP−FTP)	0.493 (±0.38)	0.349 (±0.39)	Interaction effect	1.732	0.195	0.252

FTP, Functional Threshold Power; LM, Lean Mass; RCP, Respiratory Compensation Point.

a p<0.05 (compared with RCP for the same sex).

b p<0.05 (compared with men for the same test).

*Mean significance difference (p < 0.05) when compared to RCP. #Mean significance difference (p < 0.05) when compared to men. Error bars: 95% CI.

For power output relative to total body mass (W/kg), there was a significant effect of test type (*F*(1,47) = 59.804; p < 0.001) and sex (*F*(1,47) = 14.954; p < 0.001), but no significant interaction effect (*F*(1,47) = 3.554; p = 0.066; Table 3 and Fig. 3).

For power output relative to lean body mass (W/LMkg), the analysis showed a significant effect of test type (*F*(1,47) = 58.516; p < 0.001), but no significant effect of sex (*F*(1,47) = 0.417; p = 0.521) or interaction effect (*F*(1,47) = 1.732; p = 0.195; Table 3 and Fig. 3).

**Fig. 4:**
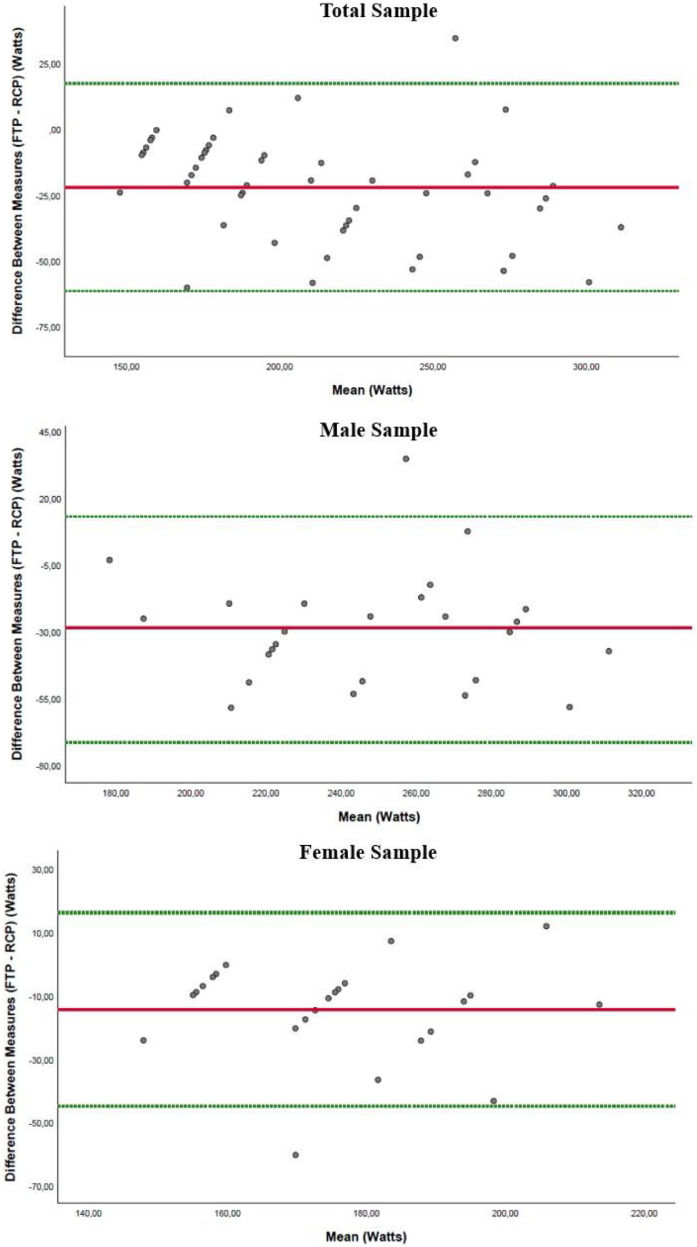
Bland-Altman plot for RCP and FTP in male and female athletes.

The Bland-Altman analysis revealed poor agreement between FTP and RCP in both male and female athletes (Fig. 4).

## Discussion

This study investigated whether the FTP test could replace the incremental test for determining power at RCP in male and female amateur triathletes. The key findings were as follows: a) FTP and power at RCP were strongly correlated considering the entire sample; b) The association between FTP and RCP was independent of sex (c) agreement between FTP and RCP power was low in both sexes; d) Power at RCP (absolute, body mass-relative, and lean body mass-relative) was higher than FTP in both sexes; and e) Men had higher absolute and body mass-relative power than women, but there was no significant sex difference in lean mass-relative power.

The authors’ hypothesis stated that although FTP and RCP would be correlated in both sexes, they would not yield equivalent values. The results partially confirmed this hypothesis. As expected, FTP and RCP were strongly correlated; however, the agreement analysis (Bland-Altman) demonstrated wide limits of agreement, indicating that the two measures are not interchangeable at the individual level. Furthermore, contrary to the exploratory expectation regarding a potential sex effect, the regression interaction term (FTP × sex) was not significant, indicating that the relationship between FTP and RCP does not differ between males and females.

Prior studies, predominantly on male athletes, have also reported strong associations between FTP and metabolic thresholds in recreational, amateur, and well-trained athletes. For instance, Barranco-Gil et al. found a strong FTP-RCP correlation (ranging from 0.86 to 0.93 in male cyclists, depending on the warm-up protocol).^10^ In the same direction, Jeffries et al. observed a strong correlation (*r* = 0.79) between FTP and power at the lactate threshold in male cyclists.^20^ In a more recent study, McGrath et al. reported a significant correlation (*r* = 0.87) between FTP and lactate threshold power in a mixed group of 50 men and 20 women,^21^ however, they did not analyze sex differences. Importantly, unlike previous studies, the present study examined men and women separately and statistically tested the sex × FTP interaction. No sex effect was detected, demonstrating that the FTP–RCP relationship does not differ between sexes. Given the historical male dominance in exercise science research, these findings help bridge the gender gap and contribute to a more inclusive understanding of physiological differences.

Although FTP and RCP were correlated, Bland-Altman plots revealed poor agreement due to wide limits of agreement and dispersion. This suggests that despite their association, FTP and RCP should not be used interchangeably for either sex. Similar findings have been reported in studies focusing on male athletes,^20^^,^^22^^,^^23^ and in mixed-sex studies,^21^^,^^24^ reinforcing the need for caution when substituting FTP for RCP in performance assessments, particularly for female athletes.

Absolute power (W) was lower for FTP than for RCP in both men and women, showing that the FTP test systematically underestimates RCP power. This aligns with Wong et al., who showed that exercising at or slightly above FTP was insufficient to reach V̇O_2_max, suggesting that FTP falls within the heavy domain intensity and does not represent the maximal metabolic steady state.^25^

The difference between FTP and RCP was significant in both men (29.21±21.49 W) and women (14.43±15.58 W), as shown in Table 2. This reinforces the findings displayed in the Bland-Altman plot, indicating that the two measures should not be used interchangeably for either sex, given the substantial magnitude of the difference in power output between FTP and RCP. In practical terms, this discrepancy may lead to different training intensity zone prescriptions. Sitko et al. reported no significant difference between FTP (289±50 W) and RCP power (287±42 W) in male road cyclists.^22^ Similarly, McGrath et al. found no difference between FTP and lactate threshold power in highly trained athletes.^24^ In contrast, Valenzuela et al. observed that FTP was lower than lactate threshold power in recreational cyclists, whereas the two values were comparable in well-trained athletes, suggesting that training status may influence the agreement between FTP and metabolic thresholds.^23^ Jeffries et al., however, reported significant differences between FTP and multiple lactate threshold methods in well-trained male cyclists.^20^ Direct comparisons among studies examining the relationship between FTP and physiological thresholds are limited by substantial methodological heterogeneity. Protocols differ in warm-up duration and structure (testing modality (laboratory ergometers^20^^,^^23^^,^^24^ vs. own bicycles mounted on smart trainers),^22^ and athlete characteristics, ranging from recreational[22,23] to highly trained cyclists.^20^^,^^24^ In addition, sex representation is inconsistent, with some studies including only men[20,23] and others adopting mixed samples without sex-specific analyses.^22^^,^^24^ These methodological discrepancies likely contribute to variability in reported associations between FTP and laboratory-derived thresholds and should be considered when interpreting between-study comparisons.

Overall, despite considerable heterogeneity across studies, which may influence the level of agreement observed between FTP and RCP power, these findings align with the evidence indicating that FTP and RCP (or lactate threshold) often differ. This suggests that relying on FTP as a surrogate for RCP may lead to an underestimation of true threshold intensity.

Regarding the differences between sexes, men demonstrated higher absolute power in both FTP and power at RCP compared with women, which aligns with established physiological differences ‒ men generally have greater muscle mass and strength.^26^ This sex-based disparity was also evident when power was adjusted for body mass, as women have a higher percentage of fat mass, which negatively affects power output.^26^ These differences emerge during puberty when testosterone levels, which drive muscle mass and strength gains, increase up to 30-times in boys but remain low in girls.^27^ In contrast, estradiol levels rise in girls but have a minimal influence on muscle mass development.^27^

However, when power output was normalized to lean body mass, no sex differences were observed. This finding suggests that the difference in absolute power between men and women in the present sample is largely explained by differences in lean mass. However, the present study was not designed to evaluate physiological mechanisms underlying power generation, and therefore, no conclusions can be drawn regarding neuromuscular, hormonal, architectural, or metabolic contributors.

### Limitations and strengths, practical applications

This study utilized the Lode cycle ergometer, a gold-standard device in cycling research, to assess FTP and RCP-associated power. The FTP test was conducted in linear mode, requiring athletes to adjust their cadence to modify power output, which may introduce bias. Although this methodology has been used in previous studies,^28^ testing on a standardized ergometer rather than the athletes’ own bicycles could also influence results. The warm-up protocol used in the FTP test was adapted from the original format to improve standardization under laboratory conditions. Although justified from a methodological standpoint, this modification may limit direct comparability with studies that employ the traditional FTP protocol and cyclists who follow the standard test in real-world training. The study’s strengths include the separate analysis of male and female athletes and the use of a validated ergometer, ensuring reliable and comparable data. From a practical standpoint, although the FTP test offers clear advantages due to its simplicity, accessibility, and feasibility as a field test, coaches and athletes should be aware that FTP consistently underestimates RCP. The 95% limits of agreement exceed ±40 W, which is wider than the typical separation between training intensity zones. Therefore, using FTP to prescribe RCP-based zones may lead to substantial errors in individual athletes.

## Conclusion

While FTP and power at RCP are correlated, the Bland–Altman analysis demonstrated wide limits of agreement for both sexes, indicating that FTP and RCP are not interchangeable. The magnitude of the limits of agreement indicates that substituting FTP for RCP may lead to different training-intensity zone prescriptions. In addition, the FTP-RCP relationship does not differ by sex. Finally, although men showed higher absolute and body-mass-relative power, lean-mass-relative power was similar between sexes, suggesting that differences in absolute power were largely explained by differences in lean mass.

## Abbreviations

ANOVA, Analysis of Variance; BM, Body Mass; DXA, Dual-Energy X-Ray Absorptiometry; FTP, Functional Threshold Power; HRmax, Maximal Heart Rate; LMkg, Lean Body Mass; RCP, Respiratory Compensation Point; RER, Respiratory Exchange Ratio; V̇CO_2_, Carbon Dioxide Production; V̇E, Minute Ventilation; V̇O_2_max, Maximal Oxygen Uptake; W, Power Output; W/BM, Power Output Relative to Body Mass; W/LMkg, Power Output Relative to Lean Body Mass.

## Authors’ contributions

Conceptualization: P.E., M.S.A.; Data curation: P.E., L.V., V.R.A.S.; Formal analysis: P.E., L.V., V.R.A.S.; Funding acquisition: M.S.A.; Investigation: P.E.; Methodology: P.E.; Project administration: M.S.A.; Supervision: M.S.A.; Validation: C.A.B.L.; Visualization: C.A.B.L.; Roles/Writing-original draft: P.E., M.S.A., K.W., B.K., T.R.; and Writing-review & editing: P.E., M.S.A., C.A.B.L., K.W., T.R., B.K.

## Data availability statement

The datasets generated and/or analyzed during the current study are available from the corresponding author upon reasonable request.

## Declaration of competing interest

The authors declare no conflicts of interest.
